# The clinical prospects and challenges of photothermal nanomaterials in myocardium recovery after myocardial infarction

**DOI:** 10.3389/fbioe.2024.1491581

**Published:** 2024-10-29

**Authors:** Jiali Yang, Jian He, Tian Yue, Haifeng Pei, Shiqiang Xiong, Yue Tang, Jun Hou

**Affiliations:** ^1^ School of Life Science and Engineering, Southwest Jiaotong University, Chengdu, China; ^2^ Department of Cardiology, Chengdu Cardiovascular Disease Research Institute, The Third People’s Hospital of Chengdu, Chengdu, China; ^3^ Department of Cardiology, The General Hospital of Western Theater Command, Chengdu, China

**Keywords:** myocardial infarction, photothermal nanomaterials, clinical applications, thermal effect mechanism, myocardial repair

## Abstract

The high morbidity and mortality rates associated with myocardial infarction pose a serious threat to human health. Early diagnosis and appropriate treatment are crucial in saving the lives of patients. In recent years, nanomaterials-based technologies have played a significant role in developing new strategies for cardiac repair, particularly in the use of photothermal nanomaterials, which show great potential in treating myocardial infarction. This review aims to describe the characteristics of photothermal nanomaterials, their effects on cardiomyocyte proliferation and angiogenesis, and the mechanism of cardiac tissue repair. This review serves as a valuable reference for the application of photothermal nanomaterials in the treatment of myocardial infarction, with the ultimate goal of expediting the translation of these treatment strategies into clinical practice.

## 1 Introduction

Cardiovascular diseases characterized by narrowing and blockage of the coronary arteries, including acute or chronic disease such as stroke, atherosclerosis and myocardial infarction, are still the leading causes of death worldwide (Khan et al., 2021; Mohammed et al., 2023). Myocardial infarction (MI), the most serious type of cardiovascular disease, is a condition in which atherosclerotic plaque breaks off and blocks off the coronary arteries, leading to myocardial ischemia and hypoxia. MI is usually caused by thrombus blocking arteries or bypass graft surgery. It is characterized by a sudden decrease in blood flow to the myocardium, which eventually leads to heart failure and death. Restoring blood flow to rescue hypoxic tissues is considered to be an effective strategy (Zhang Q. et al., 2022). Due to the relatively high mortality and incidence of MI, early diagnosis and appropriate therapeutic measures are crucial for saving lives. However, due to the complexity of its pathophysiologic conditions, the diagnosis of MI and appropriate therapeutic approaches remain major challenges (Tibaut et al., 2016).

Percutaneous coronary intervention (Ueki and Kuwahara, 2023) or intravenous thrombolytic therapy (Marto et al., 2019) can reduce the mortality of MI, but ischemia-reperfusion injury is prone to occur after opening the infarcted coronary artery, resulting in negative myocardial remodeling and cardiac insufficiency, and eventually progress to heart failure or sudden death. The current treatments have not fundamentally repaired the damaged myocardium and effectively restored cardiac function. Myocardial fibrosis after MI is one of the main causes of ventricular remodeling and cardiac dysfunction. Excessive deposition of extracellular matrix in the infarcted area and marginal area, imbalance of collagen synthesis and degradation in myocardial fibers, myocardial stiffness, decreased cardiac compliance, affecting cardiac systolic and diastolic function, leading to decreased cardiac function and even heart failure (Borrelli et al., 2021). In addition, drug therapy, including antiplatelet, antiarrhythmic and angiotensin-converting enzyme inhibitors, has also been shown to be limited in reducing infarct size and improving prognosis. Therefore, it is urgent to find an effective and alternative treatment for MI (Lv et al., 2022).

**FIGURE 1:**
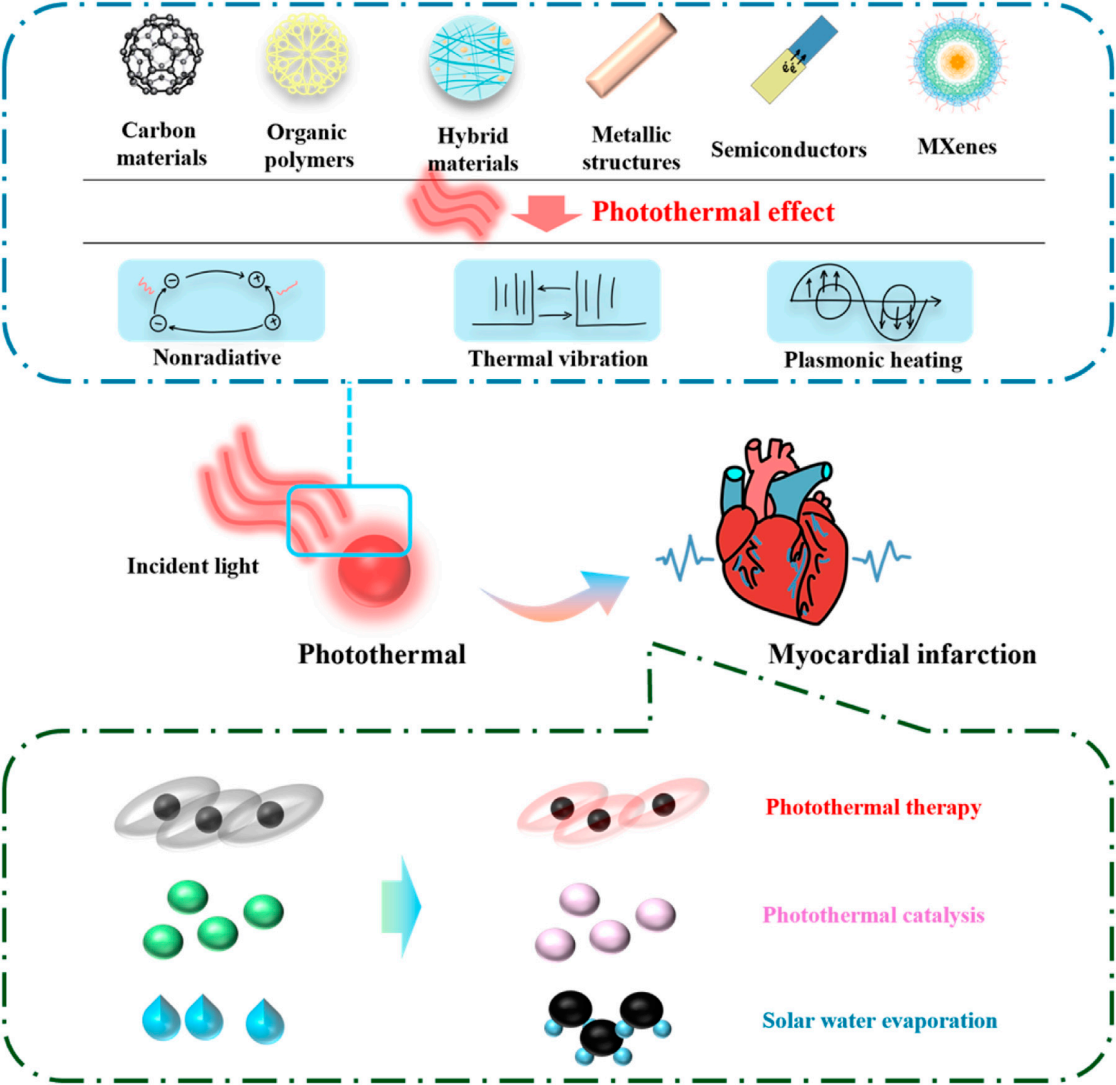
Overview of the mechanism, types and applications of photothermal nanomaterials.

Photothermal nanomaterials are a kind of nanomaterials, which are characterized by the ability to absorb light energy (usually visible light, infrared light or ultraviolet light in the electromagnetic spectrum) and convert it into heat energy. These materials usually have nanoscale structural units that can efficiently convert light energy into heat energy and have broad application prospects, such as solar thermal energy conversion and photothermal therapy. Photothermal nanomaterials include metal/semiconductor structures, carbon materials, organic polymers, two-dimensional materials and transition metal carbides/nitrides, etc. (Doughty et al., 2019). They have the characteristics of high absorption rate, rapid heating, wavelength selectivity, controllability and wide application, and have important application value in many fields (Chen et al., 2023). The mechanism, types and applications of photothermal nanomaterials are summarized in Figure 1. In the photothermal therapy of myocardial infarction, the selection of preferred nanocomposites needs to consider factors such as photothermal conversion efficiency, biocompatibility, targeting, stability, and biodegradability. The classification of common nanomaterials for the treatment of myocardial infarction and their advantages and applications are shown in Table 1.

**TABLE 1:** Common photothermal nanomaterials and their therapeutic applications.

Category	Materials	Advantages	Disadvantages	Theranostic applications	References
Metal nanomaterials	AuNPs	High photothermal conversion efficiency, good biocompatibility, easy surface functionalization	Low targeting and potential long-term toxicity	Cell imaging PTT Delivery of therapeutic cargos	Lancet and Pecht (1977), Yang et al. (2019b), Gholipourmalekabadi et al. (2017), Lee and Choi (2018)
	AgNPs			PTT Photothermal Probes Imaging	Liu et al. (2023) Di Corato et al. (2012) Vazquez-Prada et al. (2023), Lancet and Pecht (1977)
Carbon nanomaterials	Carbon nanotube	Excellent photothermal conversion performance, high specific surface area	Poor biodegradability, potential long-term toxicity	Photothermal imaging	Russier et al. (2017)
	Graphene	Good photothermal conversion efficiency and mechanical properties	Low dispersion and poor biocompatibility	PTT	Báez (2023)
Polymers	Hydrogel	Biodegradable, easy to load drugs, adjustable release characteristics	Low photothermal conversion efficiency	Drug delivery Sterilization and anti-inflammatory	Ding et al. (2022) Zheng and Xiao (2023)
	Micelle			PTT Drug release	Wang et al. (2022) Lancet and Pecht (1977)

Compared to traditional surgical treatments and drug therapies, photothermal nanomaterial therapy offers several advantages, as shown in the Table 2. After MI, damaged myocardial tissue needs to be repaired to restore cardiac function. Photothermal nanomaterials can convert light energy into heat energy by local thermal therapy, and locally heat damaged myocardial tissue. This heat therapy can promote the growth and repair of surrounding healthy myocardial cells and help restore the function of damaged myocardium (Galliot et al., 2017). Photothermal nanomaterials can be used as carriers for drug delivery systems to accurately deliver the drugs needed to repair the myocardium to the damaged area (Kim et al., 2023). Through the photothermal effect, the precise release of drugs can be achieved, the local concentration of drugs can be increased, and the repair of myocardial tissue can be promoted. The thermal effect of photothermal nanomaterials can stimulate the differentiation of stem cells and the regeneration of cardiomyocytes (Jung et al., 2021). This feature can be used to guide stem cells to differentiate into cardiomyocytes for repairing damaged myocardial tissue. Myocardial tissue repair requires not only the repair of myocardial cells, but also the promotion of new blood vessels to provide adequate oxygen and nutrition (Abo-Elfadl et al., 2016). Photothermal nanomaterials can promote the formation of new blood vessels and accelerate the repair process of myocardial tissue by stimulating the proliferation and migration of vascular endothelial cells.

**TABLE 2:** Advantages of using photothermal nanomaterials for the treatment of myocardial infarction.

Compared to other treatments	Advantages	References
vs. Drug therapy	High targeting; Strong controllability; Low drug resistance	Bao et al. (2018), Sarkar and Levi-Polyachenko (2020), Zhao et al. (2023)
vs. Interventional therapy	Non-invasive; Flexibility for multiple uses	Chen et al. (2019), Cho et al. (2021)
vs. Bypass operation of coronary artery	Low trauma	Khan et al. (2019)

In this article, we describe the effect and mechanism of photothermal nanomaterials on myocardial tissue after myocardial infarction, as well as their application in the treatment and diagnosis of MI, aiming to meet the needs of MI treatment through the innovation of photothermal nanomaterials.

## 2 The effect of photothermal nanomaterials on myocardial tissue repair after myocardial infarction

### 2.1 Preparation and properties of photothermal nanomaterials

**FIGURE 2:**
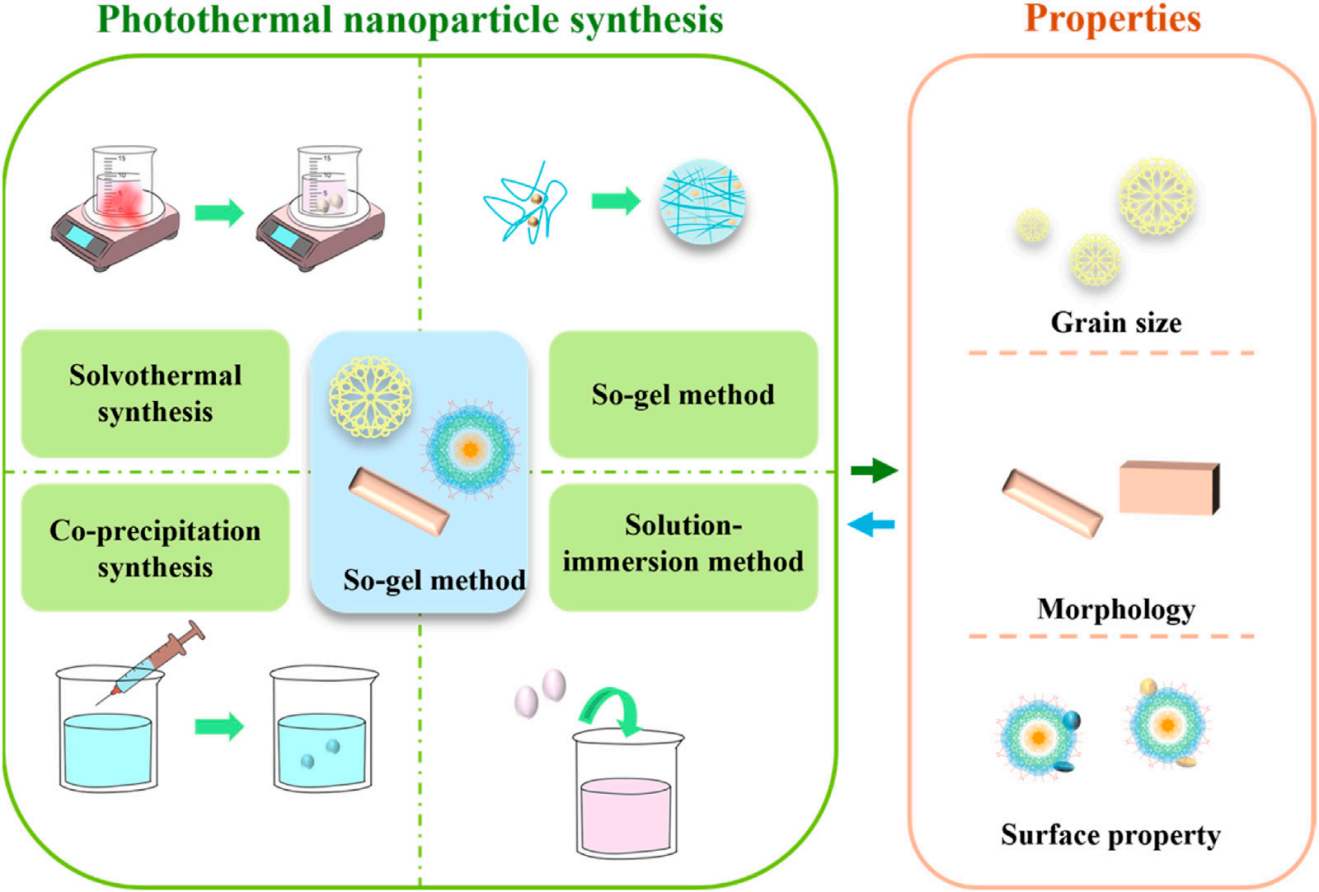
Preparation methods and properties of photothermal nanomaterials.

The preparation methods of photothermal nanomaterials are various, and the material properties determine their application direction. The preparation methods and characteristics are shown in Figure 2.

#### 2.1.1 Preparation and selection of photothermal nanomaterials

The preparation methods of photothermal materials mainly include solvothermal synthesis (Liu et al., 2019), so-gel method (Zdravkov et al., 2021), co-precipitation method (Zeng et al., 2022) and solution-immersion method (Jiang et al., 2022). The solvothermal synthesis is a method of synthesis under high temperature and high pressure conditions of the solvent. In this method, the solvent is not only a reaction medium, but also can regulate the temperature, pressure and solubility of the reaction, thereby controlling the morphology and structure of the nanomaterials. This method is often used to synthesize nanomaterials with high quality and high crystallinity. Sol-gel method is a method of converting solution or colloid into solid materials (Bokov et al., 2021). In this method, the precursor of the solution forms a gel by hydrolysis and polycondensation, and then solid materials are obtained by drying and heat treatment. This method can prepare various oxides, silicates and other nanomaterials, and can control the pore structure and surface properties of the materials. The co-precipitation synthesis usually refers to the co-precipitation of the solutes in two or more solutions to form solid particles in the solution by mixing two or more solutions (Li et al., 2017). Solution-immersion method is a common method for preparing nanocomposites, which usually includes suspending nanoparticles in a solvent and then depositing nanoparticles onto the surface of the substrate by immersion (soaking the substrate material to be treated in a nanoparticle solution) (Zhang et al., 2018).

The heart is primarily composed of myocardial tissue, which has a relatively slow rate of cell regeneration (Hesse et al., 2018). Therapeutic materials are typically in contact with blood or implanted in the tissues surrounding the heart. When using photothermal materials for to treat MI, it is important to consider their biocompatibility. This means paying attention to whether the material interacts with biological tissues in a way that could cause adverse reactions (Hong et al., 2023). Materials with good biocompatibility typically do not cause cell toxicity or immune reactions, and they can also prevent the formation of blood clots, making them safer for use in living organisms.

#### 2.1.2 Physical properties and biocompatibility of photothermal nanomaterials

The physical properties of photothermal nanomaterials include parameters such as particle size, morphology, and surface characteristics. The shape of nanoparticles can affect their application in myocardial infarction. For example, nanomaterials with needle-like and rod-like structures may help to improve the targeting of deep myocardial cells through the gap of myocardial tissue. Spherical nanoparticles may be easier to reach the infarct area through blood circulation, but may not penetrate the tissue as effectively as needle-like structures (Adnan et al., 2016). Core-shell nanoparticles can be designed to encapsulate photothermal conversion materials in the core, while the shell provides biocompatibility and targeting ligands. Such a structure can optimize the generation and transfer of heat. The particle size of nanomaterials refers to their dimensions on a nanoscale. The size of the particles can significantly impact the optical, electrical, and thermal properties of the materials (Mbalaha et al., 2023). Generally, smaller particles have larger surface-to-volume ratios, contributing to enhanced light absorption and thermal conversion efficiency. Morphology describes the external shape and structure of nanomaterials. The morphology of nanomaterials has a considerable influence on their performance in photothermal conversion. For instance, the choice of morphology, such as nanowires, nanoparticles, or nanosheets, can modulate the optical absorption and scattering properties of the material, thereby affecting its photothermal conversion efficiency (Tee et al., 2021). The surface characteristics of nanomaterials play a crucial role in their reactivity and catalytic performance in photothermal conversion (Cheng et al., 2020). Surface characteristics encompass surface energy levels, active surface sites, surface structure, etc., Optimizing surface properties can improve the interaction between photothermal nanomaterials and light or heat, enhancing their potential applications in the field of photothermal conversion. The manipulation and optimization of these physical properties represent important directions in the research of photothermal nanomaterials, aiming to achieve more efficient photothermal conversion outcomes. The photothermal conversion efficiency determines the ability of nanomaterials to effectively convert light energy into heat energy after absorbing light energy, thus directly affecting the therapeutic effect and the safety of surrounding tissues. By measuring the temperature rise curve, the conversion efficiency can also be tested by infrared thermal imaging technology, which can monitor the heat generated by nanomaterials under light and its distribution in real time (Gu and Zhong, 2023).

When using photothermal nanomaterials in the biomedical field, it is crucial for them to have good biocompatibility in order to avoid unnecessary toxicity and immune reactions. The selection of base materials with superior biocompatibility is key. Metals, oxides, and biodegradable materials are commonly used for photothermal nanomaterials, but surface modification and functionalization are often necessary. Appropriate functional groups and biocompatible coatings can reduce non-specific binding with the biological system, decrease immune system activation, and enhance biocompatibility (Wu et al., 2020). Ensure that nanomaterials can be gradually removed from the body through metabolic pathways, avoid long-term accumulation in important organs such as liver, spleen, kidney, and reduce potential organ toxicity. Another approach is to design core-shell structures, where a biocompatible outer shell is coated onto the surface of the core nanomaterial. This helps improve material stability, reduce toxicity, and simultaneously enhance overall biocompatibility by leveraging the biocompatibility of the outer layer material (Kyriakides et al., 2021; Singh and Bhateria, 2021).

### 2.2 Applications of photothermal nanomaterials in myocardial infarction model

The application of photothermal nanomaterials in MI includes promoting angiogenesis, improving arrhythmia and drug delivery.

#### 2.2.1 Establishment method of rat myocardial infarction model

Using animal models to study the pathophysiological mechanisms of cardiovascular diseases and their complications is a common and important experimental approach for researchers. Evaluating potential new treatment options and intervention measures through disease animal models is a crucial step in preclinical research. Common experimental animals for MI models include rats, rabbits, and experimental pigs. Rats are often chosen due to their pure strains, low intra-group variability, cost-effectiveness, easy maintenance, minimal collateral circulation, ease of successful modeling, and simplicity of surgical procedures. The rat MI model is a frequently utilized animal model in cardiovascular disease research. There are various methods for preparing MI models, including inducing myocardial ischemia-reperfusion injury, cause direct physical or chemical damage to the myocardium, or inducing high-risk factors for MI. These methods involve permanently ligating the coronary artery and its branches, causing thrombotic occlusion, injecting drugs into the myocardium, or implementing specific dietary methods such as high-fat or high-sugar diet.

Coronary artery ligation is a commonly used method for creating models of MI. In this approach, one or more coronary arteries are partially or completely ligated through surgical procedures, thereby interrupting the blood supply to a portion of the heart and causing ischemia and infarction in the corresponding region (Kainuma et al., 2017). By mimicking the occurrence of MI, this technique allows for the creation of infarcted and ischemic zones, making it a valuable tool for studying the mechanisms and treatment of MI (Lugrin et al., 2019). It is important to note that the establishment of an animal model for MI involves surgical procedures, which can cause certain trauma to the animals. Therefore, strict adherence to ethical principles is crucial when conducting such experiments to ensure the welfare of the animals (Kumar et al., 2016).

#### 2.2.2 The administration method and dose selection of photothermal nanomaterials

Photothermal nanomaterials can be delivered through various administration routes, including intravenous injection and local injection (Zhang P. et al., 2022). Through intravenous injection, these materials can be transported into patient’s circulatory system, allowing for distribution throughout the body and generating a heat effect in the targeted area. This method is suitable for systemic treatment or delivery through the bloodstream, such as photothermal therapy and cancer treatment (Smilowitz et al., 2018). Alternatively, in some cases, photothermal nanomaterials can be directly injected into the target area through local injection. This can be done into the myocardium, inflamed sites, or other localized areas requiring treatment. The advantage of local injection is the ability to deliver the drug more directly and precisely to the targeted tissue, reducing the impact on healthy tissues (Akbari et al., 2022). Other administration routes include oral administration (Chen et al., 2020), subcutaneous injection (Lancet and Pecht, 1977), intramuscular injection (Terentyuk et al., 2009), inhalation (Wang et al., 2023) etc. The choice of administration route depends on the characteristics of the drug, the patient’s condition, and the specific requirements of the treatment.

The selection of appropriate dosage for photothermal nanomaterials is crucial in ensuring both the effectiveness and safety of treatment. This decision must take into account the distribution and metabolic kinetics of nanomaterials within the body, as well as the specific requirements of the application. For instance, nanomaterials used for solar absorption may need to be evenly dispersed over a larger area, while those used for MI treatment may require a high concentration in a localized area (Tang et al., 2023). Additionally, the performance of photothermal nanomaterials is heavily influenced by lighting conditions. Therefore, when determining the dosage, factors such as the material’s absorption spectrum for specific wavelength of light and the intensity of light must be carefully considered. It is also important to note that different photothermal nanomaterials possess unique characteristics, such as absorption spectra, thermal conductivity, and stability. The dosage selection should consider these characteristics to ensure optimal performance in a specific application. Dosage selection for photothermal nanomaterials should be within a safe range to avoid adverse effects on the organism. Therefore, toxicity and biocompatibility are critical factors that must be carefully considered during the dosage selection process (Mathew et al., 2021). It is crucial to ensure the even dispersion of photothermal nanomaterials during use, and the preparation process should be appropriate. The choice of dosage also involves the dispersibility and stability of the material. When selecting the dosage for photothermal nanomaterials, it is recommended to conduct experimental studies and determine the optimal dosage range through systematic experiments and performance tests. Additionally, a comprehensive consideration of the above factors is necessary to make a reasonable choice based on the specific requirements of the application.

#### 2.2.3 Effect of photothermal nanomaterials on myocardial tissue repair after myocardial infarction

The application of photothermal nanomaterials in the repair and regeneration of myocardial tissue after MI is an emerging and promising research area. Although current research is still in its early stages, some experiments have indicated potential benefits of photothermal nanomaterials in the treatment of MI. One potential application is utilizing the heat effect of photothermal nanomaterials to promote tissue repair in the aftermath of MI through the localized release of thermal energy.

The use of photothermal materials has been shown to have a positive impact on angiogenesis, which is the formation of new blood vessels. This can be beneficial in repairing damaged myocardial tissue by improving blood supply to the affected area (Zeng et al., 2023). Additionally, the thermal effect of these materials can activate stem cells near the heart, causing them to differentiate into myocardial cells and aid in the regeneration of the damaged area (Zhang et al., 2020). Furthermore, the application of photothermal nanomaterials has the potential to reduce inflammation after MI by decreasing the infiltration of inflammatory cells and creating a favorable environment to repair (Xu et al., 2023). Ultimately, the use of photothermal nanomaterials can promote the repair and regeneration of myocardial tissue, leading to improved contractile function of the heart and minimizing damage to cardiac function.

### 2.3 The promoting effect of photothermal nanomaterials on cardiomyocyte proliferation and angiogenesis

#### 2.3.1 The effect of photothermal nanomaterials on the proliferation of cardiomyocytes

The use of photothermal nanomaterials in conjunction with laser irradiation has been shown to generate a heat effect that can elevate the temperature of surrounding tissues (Zhao et al., 2019). This rise in temperature has been linked to promoting cellular metabolism, including changes in lysosomal pH in live cells (Luo et al., 2016), protein synthesis (Chang et al., 2022), and other biochemical processes. By enhancing the progression of the cell cycle, photothermal nanomaterials can stimulate self-replication and proliferation of myocardial cells, ultimately leading to the proliferation and differentiation of cardiac cells. Additionally, studies have shown that photothermal nanomaterials can also regulate the function of stem cells (Carrow et al., 2020). This suggests that our photothermal nanomaterials may have the potential to promote the differentiation of stem cells into myocardial cells, contributing to the repair and regeneration of heart tissue. Furthermore, the promotion of angiogenesis by the photothermal effect can improve blood supply to the damaged area, providing essential nutrients and oxygen for the proliferation and division of myocardial cells (Liu et al., 2022). The photothermal effect can reduce inflammation and improve local blood flow through local heating, thereby providing a more favorable survival environment for stem cells (Shin et al., 2024). By regulating inflammation and oxidative stress, photothermal nanomaterials can provide protection for stem cells and reduce apoptosis (Cao et al., 2022).

#### 2.3.2 The promoting effect of photothermal nanomaterials on angiogenesis

Photothermal therapy can activate the proliferation and migration of vascular endothelial cells by influencing cell signaling pathways such as VEGF (vascular endothelial growth factor) (Gao et al., 2019). The proliferation and migration of vascular endothelial cells are critical steps in angiogenesis. By promoting these processes, photothermal nanomaterials can contribute to the formation of new blood vessels, enhancing blood supply. Angiogenesis can not only provide nutrition for damaged myocardium, but also create a more favorable microenvironment to support the proliferation and tissue repair of neonatal cardiomyocytes.

**FIGURE 3:**
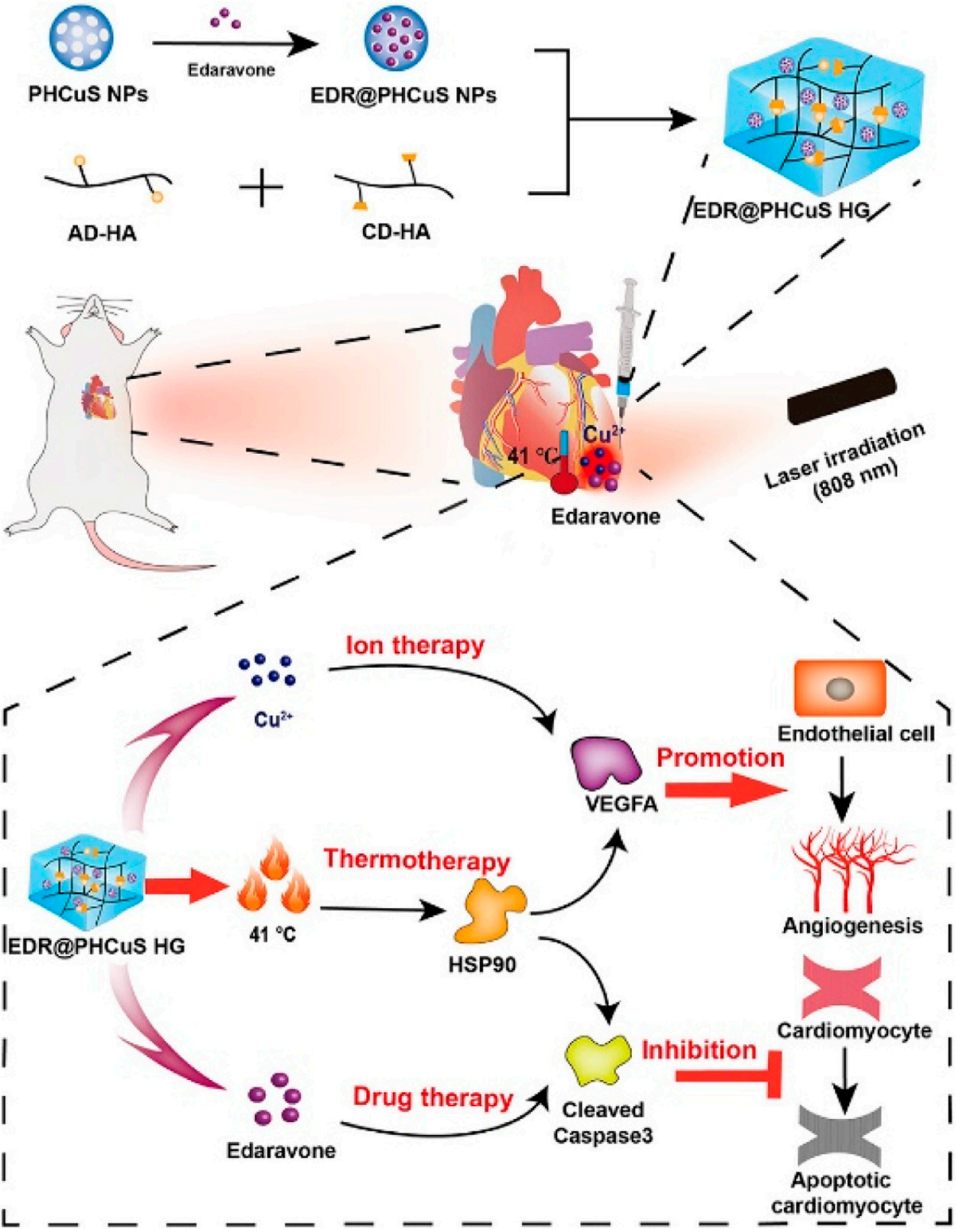
Preparation of EDR @ PHCuS HGs and its mechanism for MI treatment (Feng et al., 2023).

Ion therapy, specifically using silicate, strontium, and zinc ions, has been proven to be beneficial in treating MI. Additionally, copper ions, a trace element essential for the human body, have been shown to effectively promote angiogenesis in various diseases. Feng et al. (2023) loaded edaravone (EDR), a free radical scavenger, onto porous hollow copper sulfide nanoparticles (PHCuS NPs) encapsulated in hyaluronic acid hydrogel (HG), creating a multi-component hydrogel (EDR@PHCuS HG). When exposed to 808 nm near-infrared (NIR) light, the PHCuS NPs heated up to 41°C, inducing the expression of heat shock proteins. This synergistic release of copper ions further promoted angiogenesis, as illustrated in Figure 3.

### 2.4 Imaging application of photothermal nanomaterials in myocardial infarction heart

Gold nanoshells are commonly used for high-resolution *in vivo* imaging and photothermal therapy of tumors. Their large scattering cross-section in the second biological window also makes them suitable for molecular optical coherence tomography (OCT). When appropriately functionalized, gold nanoshells can be combined with clinical OCT to image myocardial injury after a heart attack. This is achieved by imparting selective affinity to gold nanoshells for inflammatory marker intercellular adhesion molecule-1 (ICAM-1) and apoptotic marker phosphatidylserine, which are two important mechanisms of myocardial damage after ischemia (Muñoz-Ortiz et al., 2021).

As a newly emerging principle in signaling transduction, sensors utilizing the photothermal effect have been effectively developed for visualizing biological signal readings. A cost-effective, heat-sensitive fiber paper, loaded with thermochromic dye and SiO_2_, has been created for visual display in a photothermal biosensing system used for the early detection of acute MI. By integrating a cascade enzyme immunoassay with the thermochromic fiber paper, a high-throughput testing platform has been established, utilizing induced temperature changes for real-time detection of the MI biomarker, cardiac troponin (Lancet and Pecht, 2022).

## 3 The mechanism of photothermal nanomaterials in myocardial tissue repair after myocardial infarction

### 3.1 Thermal effect mechanism of photothermal nanomaterials

#### 3.1.1 Photothermal conversion principle of photothermal nanomaterials

The photothermal conversion principle of photothermal nanomaterials involves the absorption of light energy and its conversion into heat. Generally, this process involves the absorption of light by photothermal nanomaterials, the generation of excited states, and the release of heat effects (Cui et al., 2023). Photothermal nanomaterials are typically composed of nanostructures with special optical properties. These structures can strongly absorb light at specific wavelengths (Li et al., 2021), and the absorbed light energy excites electrons or molecules within the material. After absorbing light energy, the electrons within the photothermal nanomaterial become excited and move to higher energy levels, creating an excited state. In this state, the electrons or molecules undergo non-radiative recombination, converting the excess energy into heat. This process involves the transfer of energy from the excited state to surrounding atoms and molecules through collisions or other processes, resulting in a localized increase in temperature (Lu and Wang, 2022). As heat energy, the temperature around the temperature surrounding the photothermal nanomaterial rises also rises. When evaluating the local thermal effects in tissues during the treatment of photothermal nanomaterials, the key is to accurately monitor temperature changes, tissue reactions, and the biological effects of thermal effects through various technical means such as thermal imaging, magnetic resonance thermal imaging, and photoacoustic imaging.

**FIGURE 4:**
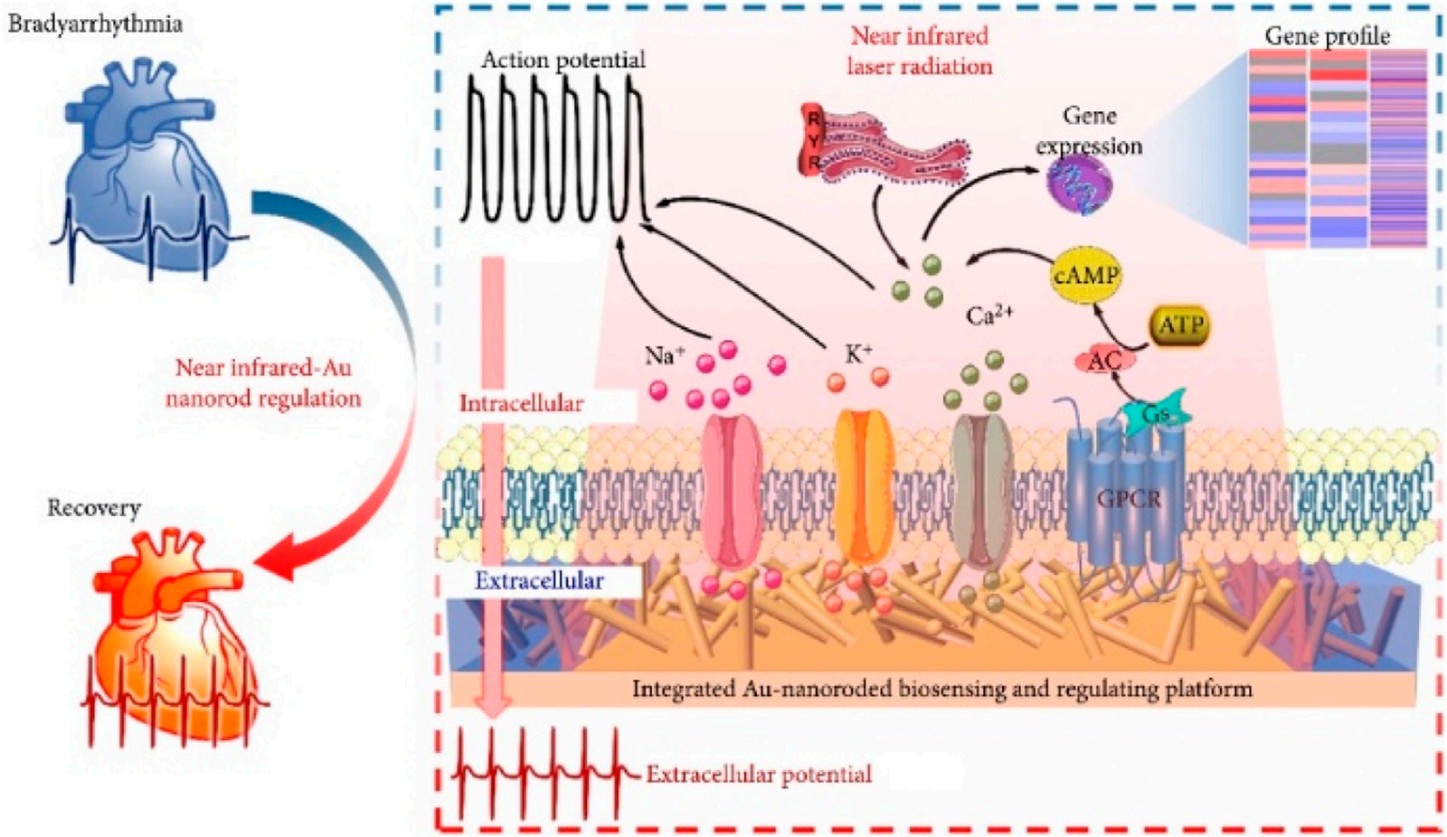
An integrated gold nanorod biosensor and regulation platform for the study of *in vitro* photothermal therapy of cardiac bradyarrhythmias (Fang et al., 2022).

Gold nanorods are a type of gold nanoparticles that have a rod-like shape. They possess photothermal effects due to the presence of two distinct absorption bands, namely transverse and longitudinal surface plasmon resonances. These resonances are caused by the presence of free electrons in the visible and near-infrared regions. The ability of gold nanorods to absorb near-infrared light makes them ideal for *in vivo* applications, including imaging and photothermal therapy. Fang et al. (2022) developed an integrated gold nanorod biosensing and modulation platform to investigate the use of photothermal treatment for *in vitro* arrhythmias, as shown in Figure 4. The Au nanoelectrode array can gently accumulates enough heat after radiation and maintains a longer repair time. mRNA sequencing revealed a significant increase in differentially expressed genes (DEGs) in cardiac cells after photothermal modulation, mainly involving ion channel genes, transport protein genes, and Ca^2+^ regulation protein genes. This establishes a promising integrated biosensing and modulation platform for the photothermal treatment of *in vitro* chronic arrhythmias, providing reliable evidence for the use of photothermal modulation of cardiac cells in clinical cardiac research.

**FIGURE 5:**
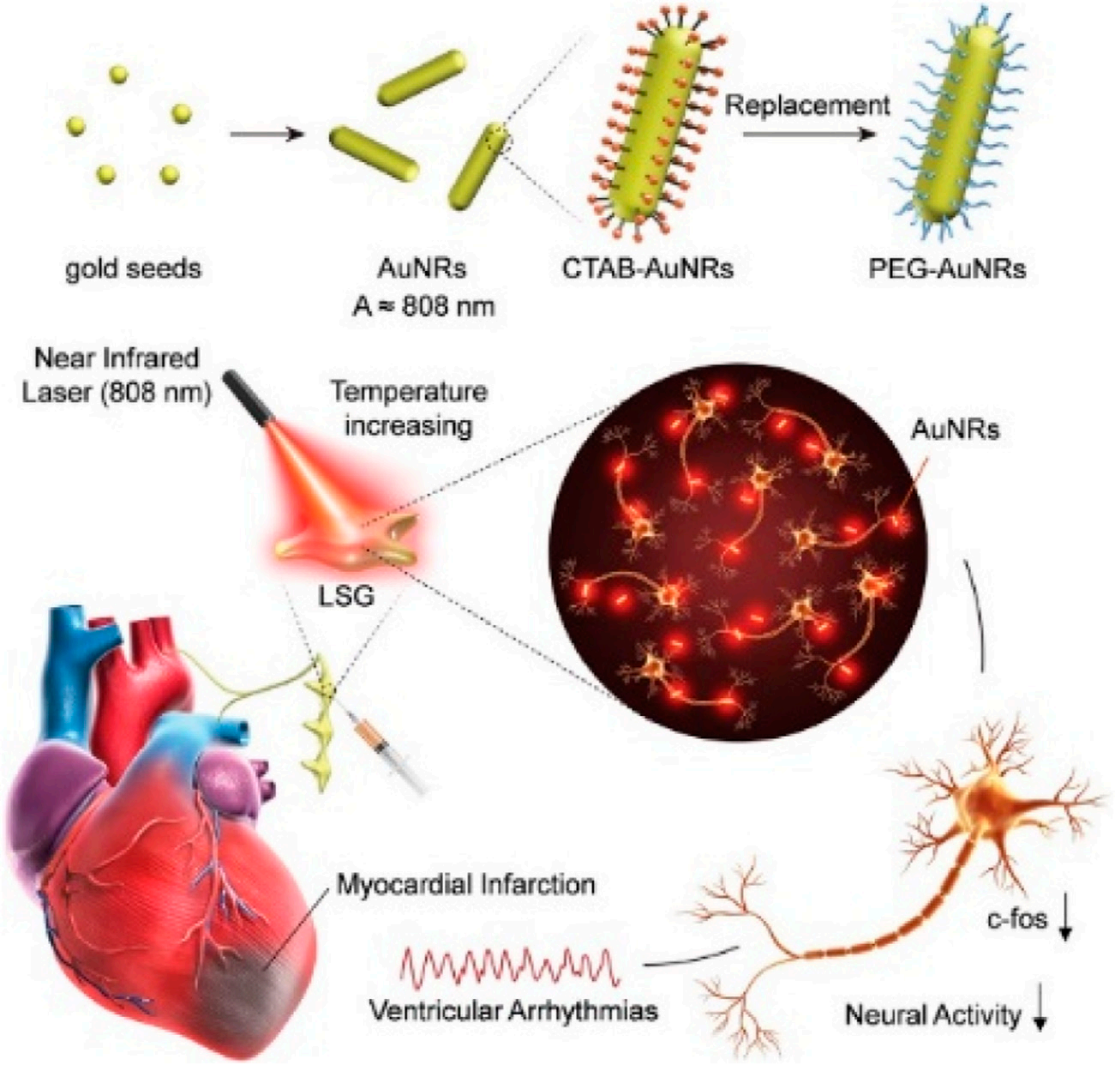
Photothermal nerve inhibition of PEG-AuNRs (Ye et al., 2019).

Ye et al. (2019) precisely controlled gold nanorods (AuNRs) to inhibit the function and neural activity of the left stellate ganglion (LSG), thereby improving myocardial ischemia-induced arrhythmias, as shown in Figure 5. Near-infrared-sensitive gold nanorods, as a biocompatible and highly efficient photothermal transducer, were employed due to their ability to convert near-infrared light into heat through surface plasmon resonance response, inhibiting neural activity through the photothermal effect of AuNRs.

#### 3.1.2 The thermal effect of photothermal nanomaterials repairs myocardial tissue

Myocardial cells rely on the delivery of oxygen and nutrients through the vascular system to maintain the heart’s normal operation. Thus, blood vessels are crucial for cells to obtain nutrition and oxygen, and the inhibition of angiogenesis significantly impacts heart repair after MI (Hu et al., 2022). Therefore, improving vascular regeneration at the infarct site is of great significance for the repair of the damaged heart after MI. Photothermal nanomaterials can promote angiogenesis by generating local heat effects. This is because the localized temperature increase can enhance blood flow, induce blood vessel dilation, and activate relevant signaling pathways for angiogenesis. In the repair of myocardial tissue, adequate blood supply is essential for providing sufficient oxygen and nutrients, aiding in promoting new blood vessel formation and myocardial repair. The thermal effect can enhance the metabolic activity of myocardial cells, promoting cell proliferation and repair. Moderate thermal effects can stimulate the synthesis of collagen, contributing to the formation of connective tissue that supports the structure of myocardial tissue. This helps enhance the structural stability of the damaged area and promotes myocardial repair. Additionally, photothermal nanomaterials may induce the expression of matrix metalloproteinases (MMPs) that help in degrading excess extracellular matrix (ECM) (Yang et al., 2019a), which can limit the formation of excessive scar tissue.

### 3.2 The biological effect mechanism of photothermal nanomaterials

#### 3.2.1 Interaction between photothermal nanomaterials and cells

Photothermal nanomaterials can undergo physical and chemical interactions with cells. This interaction can influence the physiological and metabolic status of cells, with specific effects depending on the properties, shapes, surface characteristics, and distribution within myocardial tissue of the nanomaterials. The surface characteristics of materials play a crucial role in their interactions within the biological system. The surface properties of nanomaterials can affect their interaction with myocardial cells by influencing cell adhesion, uptake, and signal transduction.

Gao et al. (2018) combined copper sulfide nanoparticles with an antibody targeting TRPV1 to create a photothermal switch for TRPV1 signal transduction in vascular smooth muscle cells (VSMCs). Upon photothermal irradiation, the localized temperature increase opened the thermosensitive TRPV1 channel, leading to Ca^2+^ influx, activation of autophagy, and inhibition of foam cell formation. This approach shows potential as a therapeutic tool for treating atherosclerosis. In a similar manner, laser stimulation mediated by gold nanoparticles has been shown to induce the contraction of myocardial cells (Gentemann et al., 2017). This method utilizes the local photothermal effect generated by the interaction between laser light and gold nanoparticles to stimulate cell contraction.

Gholami Derami et al. (2021) demonstrated that polydopamine (PDA) nanoparticles, as photothermal converters, can be reversibly regulated for myocardial cell modulation. PDA nanoparticles can finely control the regulation of excitability of cells by altering excitation power density and irradiation duration. Under optimal conditions, they proved the reversible enhancement (twofold) of the beating rate of myocardial cells.

Photothermal nanomaterials generate thermal effect by excitation of external light source, and this local heating can increase the local temperature of myocardial infarction site. Moderate heat stress can activate a series of cell signaling pathways (such as the heat shock protein pathway). Local thermal effects can induce the expression of HSPs, which help cells cope with injury and stress, promote cell proliferation, inhibition of apoptosis, and repair of damaged tissues (Fang et al., 2022; Shu et al., 2024).

#### 3.2.2 Effects of photothermal nanomaterials on inflammatory response and immune regulation

Photothermal nanomaterials have the potential to regulate inflammatory responses and immune functions. These materials exhibit antioxidative properties, helping to reduce inflammation induced by oxidative stress. By inhibiting oxidative stress reactions, they impact the activity of immune cells. Photothermal nanomaterials can also serve as drug carriers, influencing inflammatory responses by regulating drug release. Through achieving targeted and controlled drug delivery, they contribute to the precise modulation of immune functions.

Research has shown that gold nanorods can be successfully delivered to inflamed joint tissues in a rat model of adjuvant-induced arthritis. However, there is currently no information on the accumulation of gold nanorods in inflamed myocardium. In order to address this gap, Higuchi et al. (2019) conducted a study to investigate the delivery process of gold nanorods to the hearts of transgenic mice with inflammatory cardiomyopathy. The results showed that PEGylated gold nanorods accumulated more in the hearts of mice with inflammatory cardiomyopathy compared to normal hearts. This accumulation was found to be proportional to the severity of ventricular hypertrophy in the failing heart. The exact mechanism of this accumulation is not yet clear, but it is possible that sustained inflammation and endothelial dysfunction in the local microenvironment of inflammatory cardiomyopathy play a role, similar to the permeability and retention effects observed in the cancer vascular microenvironment.

## 4 The challenges and prospects of photothermal nanomaterials in clinical applications

### 4.1 Safety and toxicity evaluation of photothermal nanomaterials

#### 4.1.1 Biosafety assessment of photothermal nanomaterials

The toxicity of nanomaterials is often attributed to their physicochemical properties, such as geometric shape, size, surface charge, hydrophobicity, and crystallinity. Understanding the potential toxicity of these materials is a crucial for risk assessment and safe applications. The biological safety assessment of nanomaterials typically involves a series of *in vitro* and *in vivo* toxicity experiments, as well as safety evaluations. *In vivo* compatibility includes blood compatibility, tissue compatibility, neurotoxicity, and other factors (Wang et al., 2019). Traditional, low-throughput measurement methods are commonly used to assess well-known toxicity endpoints, such as the generation of reactive oxygen species, cell toxicity (cell death/cell viability), and DNA damage (Fadeel et al., 2018). To ensure a thorough understanding of the biocompatibility of photothermal nanomaterial, it is necessary to conduct a comprehensive examination of their safety, considering different doses and time points. The results of biocompatibility assessments will directly impact the clinical application and commercial prospects of these materials. In addition, if we want to consider the safety of human body, we can preliminarily consider the use of human cell lines or primary cells to study the interaction between nanomaterials and cells, and explore the potential cytotoxicity mechanism.

#### 4.1.2 Toxicity assessment and side effect analysis of photothermal nanomaterials

The assessment of toxicity for photothermal nanomaterials must take into account their metabolism (Pantic et al., 2019) and excretion kinetics *in vivo*. It is crucial to study the metabolic pathways of these nanomaterials within a biological system, determine if any metabolic transformations occur, and characterize the nature of the resulting metabolites. This is necessary in order to identify any potential toxic substances or metabolites. Additionally, investigating the distribution kinetics of photothermal nanomaterials *in vivo* can provide insight into whether these nanomaterials accumulate in specific organs and if this accumulation is related to toxicity. Furthermore, researching the clearance kinetics of these photothermal nanomaterials *in vivo*, including parameters such as half-life, aids in evaluating the residence time of nanomaterials in the body and their potential impact on toxicity and biological effects.

Analyzing the side effects of photothermal nanomaterials is a crucial aspect of assessing their safety for clinical applications. Considering potential side effects under light conditions, including whether local temperature elevation causes discomfort, tissue damage, or inflammation, is important. Understanding whether photothermal nanomaterials induce immune responses, inflammation, and other adverse biological compatibility reactions is essential. If photothermal nanomaterials are used in drug delivery systems, analyzing whether carrying drugs and the photothermal material itself can cause adverse reactions, including drug interactions and potential toxicity, is necessary. Si O_2_ has good biocompatibility, and polymers can be designed to achieve biodegradation and reduce the risk of long-term accumulation in the body. These can be considered for large-scale clinical applications (Li et al., 2020). Adverse reactions were effectively monitored by *in vivo* imaging techniques such as blood biomarker detection, histopathological analysis, liver and kidney function analysis, etc.

## 5 The clinical application prospect of photothermal nanomaterials

### 5.1 Potential application of photothermal nanomaterials in the treatment of myocardial infarction

Photothermal nanomaterials can serve as an adjunctive approach for MI treatment. Firstly, they can be utilized for local temperature elevation and angiogenesis. Photothermal nanomaterials, when exposed to specific wavelengths of light, induce localized temperature elevation. This effect helps promote angiogenesis, improving blood supply in the infarcted area. Enhanced blood circulation facilitates an adequate supply of oxygen and nutrients, promoting repair in the infarcted region. Secondly, photothermal nanomaterials can suppress inflammation and facilitate tissue repair. The photothermal effect aids in inhibiting inflammatory responses, reducing inflammation in the infarcted area. Simultaneously, moderate local temperature elevation may stimulate tissue repair processes, fostering regeneration and repair of myocardial cells. Finally, photothermal nanomaterials possess excellent optical properties, making them suitable for biological imaging. During the treatment process, they can be employed as contrast agents for precise image-guided therapy, ensuring the accuracy and safety of the treatment.

When we use photothermal nanomaterials, we need to take into account the influence of wavelength. The wavelength of light is indeed a key factor in the clinical use of photothermal nanomaterials, as it determines the extent to which the material can penetrate tissue, generate heat, and minimize collateral damage. Near-infrared light, especially in the range of 700–1,000 nm, has become the most promising choice due to its balance of deep tissue penetration and safety, and is very suitable for clinical applications such as cardiac tissue regeneration.

### 5.2 The combined application of photothermal nanomaterials and other therapeutic methods

**FIGURE 6:**
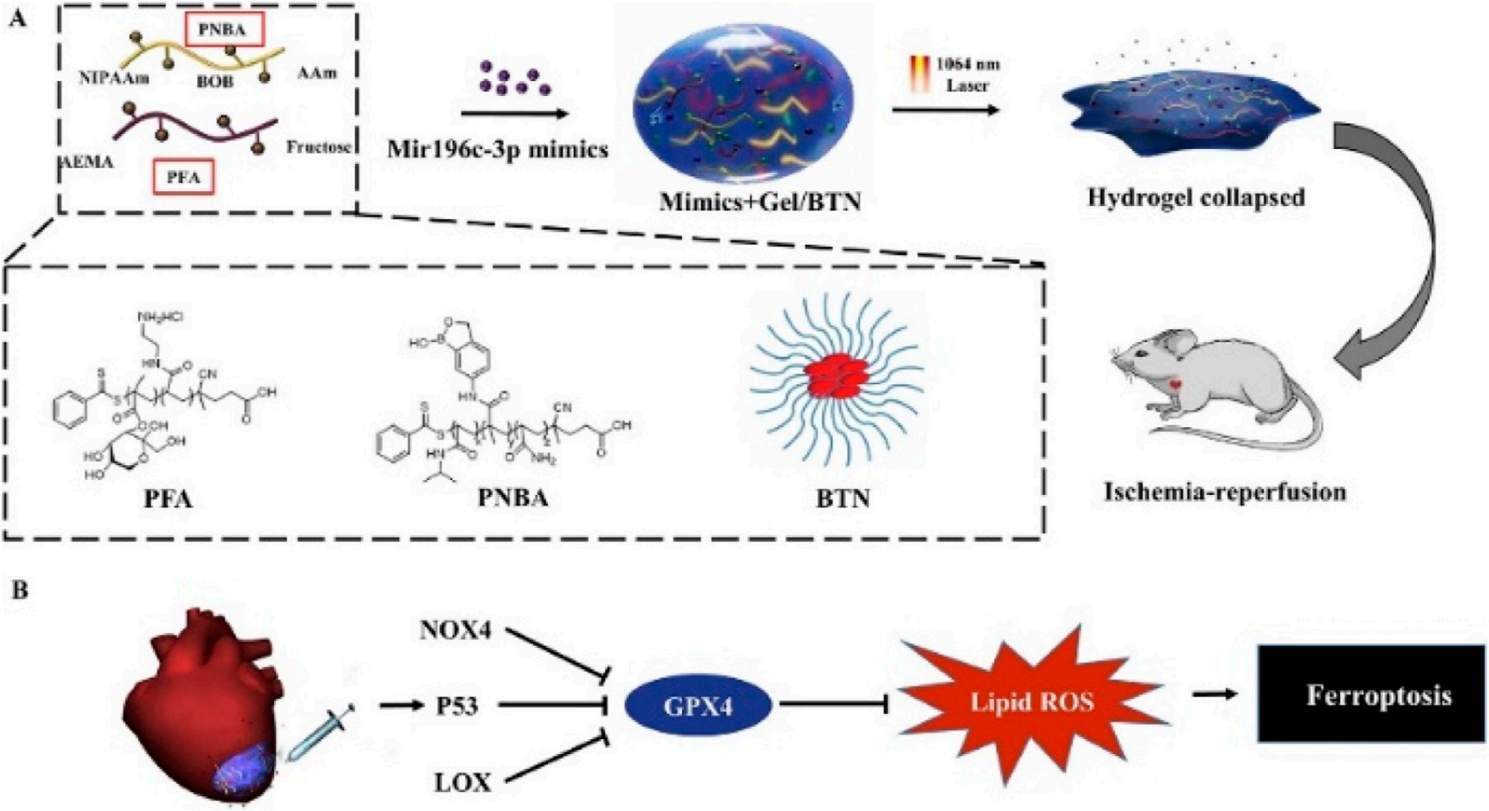
**(A)**. Preparation of hydrogel. **(B)**. The hydrogel mechanism diagram for ferroptosis treatment.

Photothermal nanomaterials can serve as effective carriers for drug delivery systems. By modulating the photothermal effect, targeted drug release can be achieved, enhancing the local concentration of drugs in diseased tissues and thereby improving therapeutic efficacy. This holds potential advantages for treating cardiac diseases such as MI. Ji et al. (2023) developed an injectable hydrogel triggered by near-infrared II (NIR-II) light, with maximal permissible exposure for deep tissue penetration and biocompatibility, for on-site treatment of ischemia-reperfusion, as illustrated in Figure 6. MiRNA and photothermal nanoparticles were co-encapsulated in a releasable hydrogel with NIR-II light-triggered release, enabling on-demand non-invasive delivery of miRNA in the local microenvironment triggered by photothermal effects.

Photothermal nanomaterials can be combined with gene therapy approaches (Liu et al., 2020). By attaching gene carriers to the surface or interior of photothermal nanomaterials, targeted gene delivery can be achieved. The photothermal effect helps enhance gene transfection efficiency (Kim and Kim, 2014), increasing cell uptake and expression of genes, thereby improving the effectiveness of gene therapy. The photothermal effect of nanomaterials can synergize with drug therapy, enhancing therapeutic outcomes. Photothermal effects enhance drug efficacy by promoting drug absorption, improving cell permeability (Sutanto et al., 2021), or optimizing the local distribution of drugs.

## 6 Conclusion and foresight

Photothermal nanomaterials have shown potential in promoting the repair and regeneration of myocardial tissue after MI. This is achieved through mechanisms such as modulating cardiac cell rhythms, promoting angiogenesis, and facilitating drug delivery. However, it is essential to note that research in this area is still in its early stages and has primarily been conducted in animal models. Before considering the use of photothermal nanomaterials in clinical treatment, thorough studies are necessary to address a series of challenges. These include assessing the material’s biocompatibility, toxicity, and long-term effects. Additionally, determining the optimal application methods and dosages in real treatment scenarios is crucial. Overall, the research on the use of photothermal nanomaterials for repairing and regenerating myocardial tissue after MI shows potential for clinical application. However, more studies are needed to confirm their safety and effectiveness and to further their use in clinical practice.

Subsequent investigations can concentrate on refining photothermal nanomaterial fabrication techniques and material choices to improve their efficacy, biocompatibility, and therapeutic outcomes. Researchers can explore novel nanomaterials with improved photothermal properties, biocompatibility, and drug delivery capabilities. By continuously improving material preparation methods and combining materials with other treatment modalities, multi-modal therapies can be achieved. Further investigation into the mechanisms of action of photothermal nanomaterials in the repair of myocardial tissue after MI is also warranted. This could involve exploring the regulatory mechanisms of angiogenesis, cellular signaling pathways, drug delivery, and the imaging of gene therapies. Integrating approaches from various disciplines such as biology, biochemistry, and immunology is crucial to comprehensively understand the mechanisms of action of photothermal nanomaterials in MI treatment. In-depth mechanistic studies can guide clinical applications and provide a scientific basis for the development of novel therapeutic methods.

Future study endeavors ought to prioritize the thorough evaluation of photothermal nanoparticles’ safety and effectiveness in medical settings. This entails assessing the drugs’ toxicity, biological reactions, efficiency of medication administration, and general patient outcomes. Evaluations of photothermal nanomaterials’ safety, biological responses, treatment efficacy, and drug delivery systems’ efficiency will help establish the viability and safety of photothermal nanomaterials’ clinical applications and lay the scientific groundwork for their broad use in the medical industry.
